# Waldenström macroglobulinemia-associated renal AL amyloidosis: a case report and literature review

**DOI:** 10.3389/fimmu.2026.1821051

**Published:** 2026-06-16

**Authors:** Jianping Zhang, Zhifeng Li, Huiping Chen, Xiaoyan Bian, Di Zhao, Zhu Lin, Tiekun Li, Yanlin Zhang

**Affiliations:** 1 Department of Nephrology, Xiamen Humanity Hospital, Fujian Medical University, Xiamen, China; 2 Department of Hematology, The First Affiliated Hospital of Xiamen University, Xiamen, China; 3 Expert Consultation Office, Jinling Hospital, Nanjing, China; 4 Nanjing Kingmed Diagnostics, Nanjing, China

**Keywords:** AL amyloidosis, chemoimmunotherapy, kidney, MYD88 L265P mutation, pulmonary infection, toxicity, Waldenström macroglobulinemia

## Abstract

Waldenström macroglobulinemia (WM) is a rare indolent B-cell lymphoma, characterized by lymphoplasmacytic infiltration of bone marrow and the existence of monoclonal IgM in circulation. Renal AL amyloidosis is an uncommon complication of WM, and its treatment is an enormous challenge to clinicians. This report describes a 71-year-old woman who presented with a 10-month history of recurrent bilateral lower extremity edema and a progressive fatigue. Physical and laboratory examinations revealed serum monoclonal IgMλ, nephrotic syndrome, moderate anemia and mild enlargement of bilateral inguinal superficial lymph nodes. Bone marrow aspiration demonstrated clonal lymphoplasmacytic infiltration, lymph node biopsy was consistent with lymphoplasmacytic lymphoma, and peripheral blood testing confirmed the presence of the *MYD88* ^L265P^ mutation. Renal biopsy confirmed the diagnosis of renal AL amyloidosis, evidenced by Congo red-positive amorphous deposits with λ light chain restriction in glomeruli, interstitium, and small arterial walls. Following multidisciplinary consultation, the patient was treated with the bendamustine plus rituximab (BR) regimen. After three cycles, the patient achieved partial hematologic response (serum IgM levels decreased from 23.7 g/L to 7.1 g/L) with slight improvement of proteinuria (urine protein excretion decreased from 18.2 g/d to 15.0 g/d). However, during the subsequent treatment, the patient developed a severe pulmonary infection that led to death. This case underscores two critical points: First, patients with WM should be routinely tested for serum and urine monoclonal free light chains, as they can exist in WM at a high proportion and can independently induce monoclonal gammopathy-associated renal diseases, including renal amyloidosis. Second, for WM patients with renal AL amyloidosis, choosing BR regimen as the first-line treatment is reasonable. However, during the treatment process, it is essential to closely monitor and actively prevent the adverse effects of this regimen, particularly severe infections which may have fatal consequences.

## 1 Introduction

Waldenström macroglobulinemia (WM) is an indolent mature B-cell lymphoma constituting <2% of B cell non-Hodgkin lymphomas, which is characterized by clonal lymphoplasmacytic infiltration in the bone marrow and the presence of monoclonal IgM (M-IgM) in the circulation (1, 2). The age-adjusted incidence of WM in the US is 0.36 per 100–000 population (3, 4). About 30% of WM patients have no clinical symptoms, but the remaining 70% are symptomatic WM. The clinical symptoms of WM are primarily caused by the following two pathogenic factors: (1) Lymphoplasmacytic infiltration (in 10–24% of cases): in patients with WM, tumor cells usually infiltrate bone marrow and may also infiltrate extramedullary tissues, including the kidneys, causing corresponding symptoms. (2) M protein-caused lesions: besides M-IgM, coexisting monoclonal free light chains (M-FLC) can also independently cause organ damage, leading to corresponding clinical manifestations (4). The incidence of WM-related renal diseases is very low. According to literature reports, it only accounted for about 3.3% in renal biopsy cases of WM (5, 6) and 3.8-7.4% in an autopsy series of WM (7). Kidney involvement in WM is relatively rare but shows a diverse range of pathological presentations. Currently, renal pathology associated with WM is primarily classified into monoclonal gammopathy-related kidney lesions and non-monoclonal gammopathy-related kidney lesions. Monoclonal gammopathy-related kidney lesions include monoclonal immunoglobulin-related amyloidosis, non-amyloid glomerulopathy, and tubulointerstitial nephropathy (8–10). Immunoglobulin light chain (AL) amyloidosis is the most common renal lesion in WM, as demonstrated in previous studies (7).

WM is an indolent and currently incurable lymphoma, yet its expanding armamentarium of therapies has significantly improved outcomes in first- and subsequent-line treatment. Current major treatment options include chemoimmunotherapy, proteasome inhibitors (PIs), BTK inhibitors, autologous stem cell transplantation (ASCT) (11). The availability of these therapeutic approaches has greatly improved survival rates, but has also introduced a range of toxicities. As a result, how to balance the goal of prolonging survival with minimizing treatment-related toxicity has become increasingly important.

Here, we report a rare case of WM-associated renal AL amyloidosis with tumor cell infiltration of bone marrow and lymph nodes. The patient achieved partial hematologic response and renal response following treatment with bendamustine and rituximab (BR), but ultimately died of severe pulmonary infection. To the best of our knowledge, there are currently no sufficient data describing the toxicities of chemotherapeutic agents, targeted therapies, or ASCT used in the treatment of WM. we briefly review some of the toxicities associated with these treatments.

## 2 Case description

A 71-year-old female patient developed progressive edema in bilateral lower extremities and fatigue for 10-month. Laboratory tests in a local hospital revealed massive proteinuria (10 g/day), hypoalbuminemia (28.7 g/L), and normal serum creatinine (57.7 μmol/L) consistent with a diagnosis of nephrotic syndrome. She had no significant medical history before. Physical examination on admission showed that blood pressure was 110/61 mmHg, bilateral superficial inguinal lymph nodes were palpable without tenderness, and palpebral conjunctiva was moderately pale. cardiopulmonary and abdominal examinations showed no abnormal findings, and both lower extremities had moderate pitting edema.

The laboratory test results were as follows: hemoglobin 84 g/L, urine protein excretion 18.2 g/d, urine RBC 1-2/HPF, serum albumin 14.3 g/L, globulin 32.2 g/L, serum creatinine 66.5 μmol/L, estimated glomerular filtration rate 79.2 mL/min/1.73m², serum IgM 23.7 g/L, IgG 1.8 g/L, IgA 0.1 g/L, serum C3 0.87 g/L, C4 0.13g/L. Serum protein electrophoresis showed a M-protein band, and the M-protein level was 15.2 g/L. Urine protein electrophoresis also showed a M-protein band, and the amount of M-protein was 5 g/d. Serum and urine immunofixation electrophoresis both revealed a monoclonal IgM lambda (M-IgMλ) band. Urine immunofixation electrophoresis which is special for Bence-Jones protein testing revealed a free lambda light chain band. Serum free light chain assay showed free kappa light chains of 13.5 mg/L and free lambda light chains of 102 mg/L, with a κ/λ ratio of 0.13. Bone marrow smear showed that 74% of mature small lymphocytes and a small number of cells with plasmacytoid features. Bone marrow flow cytometry showed B cell populations of CD19+, CD20+, CD79b+, CD23-, CD10-, CD5-, CD38- and CD138-, with lambda light chain restriction. Droplet digital polymerase chain reaction (ddPCR) of peripheral blood samples confirmed the *MYD88* ^L265P^ mutation. A biopsy of the right inguinal lymph node found low-grade B cell non-Hodgkin lymphomas, consistent with lymphoplasmacytic lymphoma. Immunohistochemistry study of lymph node biopsy tissue showed that most tumor cells expressed CD20+, CD79a+, CD5±, CD23- and CD10-, some expressed IgM+, and a few expressed CD38+ and CD138 +. Based on all the above examination results, the WM diagnosis is established.

**Figure 1:**
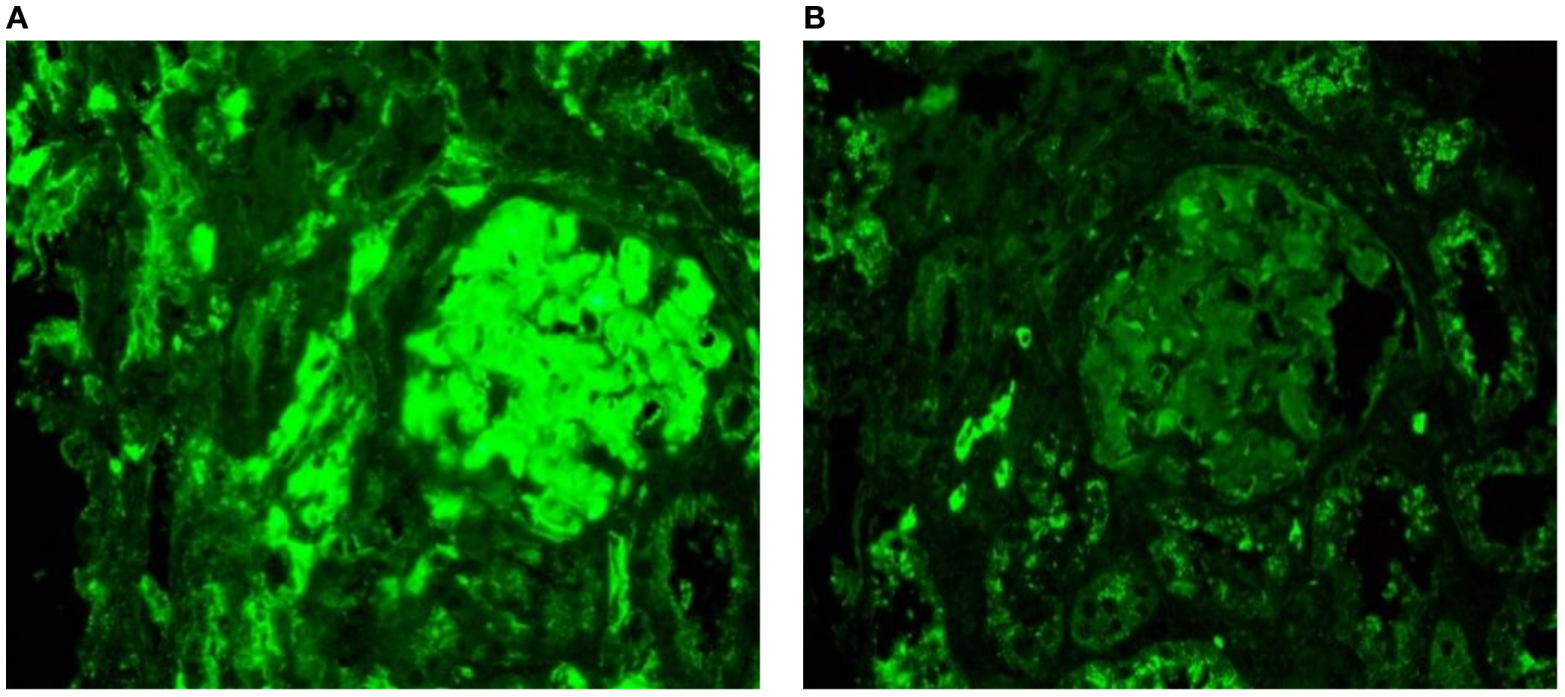
Immunofluorescence microscopic images of frozen kidney tissue. **(A)** λ light chain deposits in glomeruli and interstitium (×400). **(B)** negative κ light chain staining (×400).

**Figure 2:**
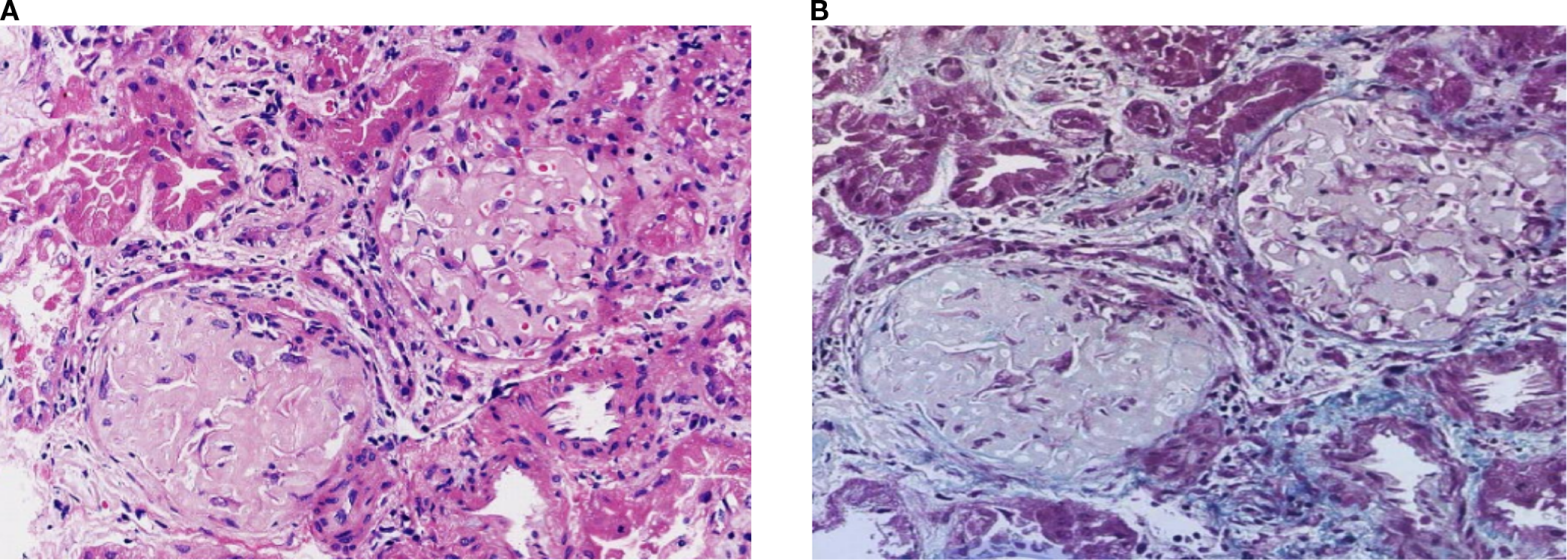
Light microscopic images of kidney tissue. Acellular and amorphous deposits in mesangial areas [**(A)** HE ×200; **(B)** Masson ×200].

**Figure 3:**
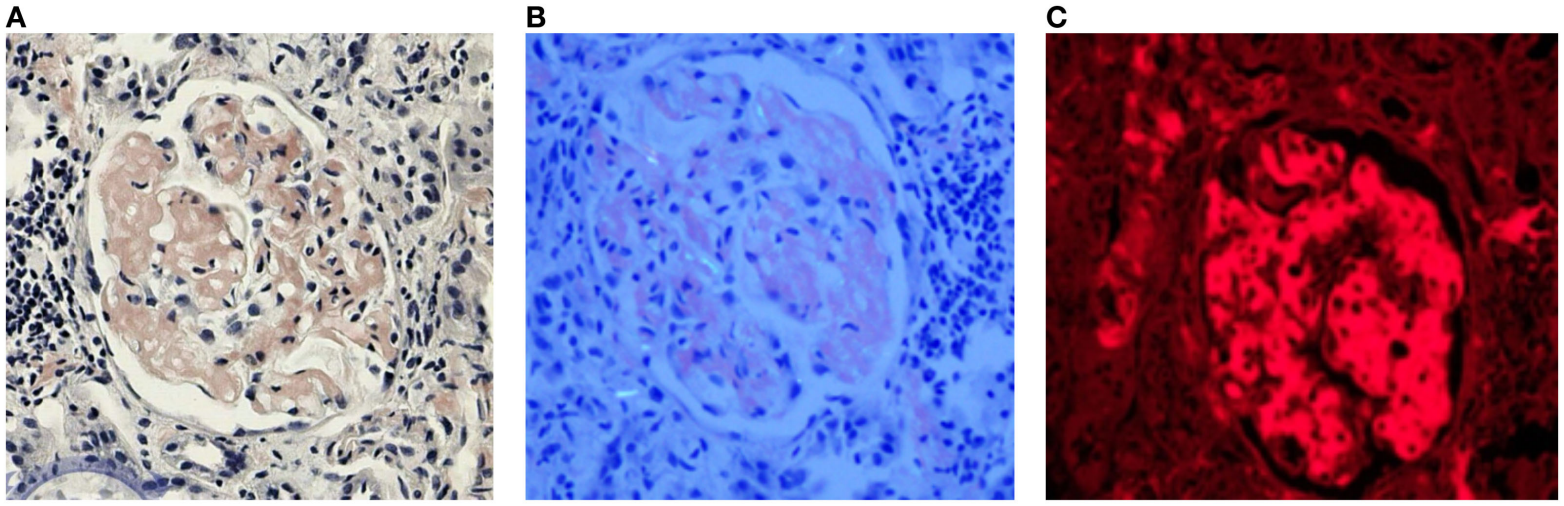
Congo red staining of kidney tissue. Congo red staining-positive deposits in mesangial areas, capillary walls, and interstitium. **(A)** Light microscopy (×400). **(B)** Polarized microscopy (×400). **(C)** Fluorescence microscopy (×400).

A renal biopsy was then performed. Immunofluorescence examination showed that lambda light chain 3+ deposited in glomeruli (mainly in mesangial areas) and interstitium, while IgG, IgM, IgA, C3, C1q and kappa light chain were all negative (**Figures 1A, B**). Light microscopy revealed that acellular, amorphous substances diffusely deposited in glomerular mesangial areas and scatted in renal interstitial tissue and small arterial walls (**Figures 2A, B**). Silver staining-positive spikes on the outer side of glomerular basement membrane were occasionally observed. There were no pseudothrombi in the capillary lumina. Congo red staining was positive in the glomeruli, interstitium and small arterial walls, showing apple-green birefringence under polarized light (**Figures 3A–C**). Electron microscopy examination could not be performed due to insufficient specimen. According to the above renal pathological manifestations combined with the patient’s clinical and laboratory findings, the diagnosis of WM-associated renal AL amyloidosis can be confirmed.

**Figure 4:**
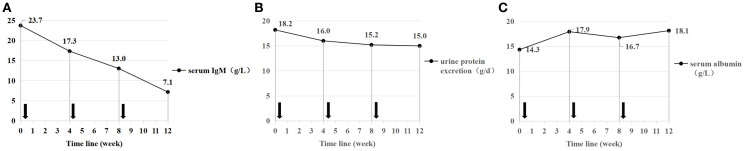
Changes in serum IgM **(A)**, urine protein excretion **(B)**, and serum albumin **(C)** levels during follow-up. Arrows denote BR sessions.

In accordance with the opinions of multidisciplinary consultation, patients began to receive BR regimen treatment (bendamustine 70 mg/m² on days 1 and 2 and rituximab 375 mg/m² on day 0. every 28 days as a cycle). After three cycles of treatment, WM had achieved a partial hematologic response (serum IgM levels decreased from 23.7 g/L to 7.1 g/L and the previously enlarged inguinal lymph nodes had markedly reduced). However, the proteinuria only got slight improvement (urine protein excretion decreased from 18.2 g/d to 15.0 g/d, and serum albumin mildly increased from 14.3 g/L to 18.1 g/L). During the subsequent treatment, the patient developed severe pulmonary infection and eventually died. The timeline of major treatments is presented in **Figures 4A–C**.

## 3 Discussion

### 3.1 Diagnosis and differential diagnosis

After hospitalization, routine inspection found that the patient’s serum IgM level increased significantly, serum IgG and IgA levels decreased markedly, along with anemia. These findings suggest that the patient may have IgM-type monoclonal gammopathy, including IgM-type malignant hematological diseases. Therefore, the patient underwent a comprehensive evaluation, including serum and urine monoclonal immunoglobulin and free light chain testing, bone marrow smear and flow cytometry analyses, pathological examination of lymph node biopsy tissue and somatic mutation detection of *MYD88*. The results of above evaluation showed that this patient had the following disease characteristics: (1) serum and urine M-IgMλ and monoclonal free light chain lambda (M-FLCλ) were both positive. (2) tumor cells infiltrated the bone marrow and lymph nodes, and their immunophenotype accorded with WM. (3) gene testing revealed the *MYD88 ^L265P^* somatic mutation. In addition, no other types of B cell lymphoma were found. Therefore, based on the above characteristics, WM diagnosis can be established (11, 12).

In the monoclonal gammopathy-associated renal diseases (MGP-RD) caused by WM, priority should be given to the following three diseases: (1) intracapillary monoclonal IgM deposits disease (ICMDD): high concentrations of M-IgM accumulate within the glomerular capillary lumen, sometimes also in arterioles and peritubular capillaries, forming pseudothrombi. When pseudothrombi severely obstruct renal microcirculation, acute kidney injury occurs clinically (13). However, no pseudothrombi were found in the glomeruli of this patient, so ICMDD can be excluded. (2) cryoglobulinemic glomerulonephritis (Cryo GN): The cryoglobulinemia complicated by Waldenström macroglobulinemia is mainly type I cryoglobulinemia, which may induce Cryo GN. Its prominent clinical manifestation is nephritic syndrome. Pathological examination shows proliferative glomerulonephritis with intracapillary pseudothrombi, and immunofluorescence reveals deposition of M-IgM and complement C3 in the capillary walls, mesangial areas, and pseudothrombi (5, 7). This patient’s presentation did not match Cryo GN (3). renal amyloidosis: Among WM-related amyloidosis, AL amyloidosis is the most common and often involves the kidney. Renal AL amyloidosis, in contrast to the two aforementioned renal diseases caused by M-IgM, is induced by β-misfolded M-FLC. About 76.5% of WM patients have M-FLC in circulation, which coexist with M-IgM (14) and can independently cause renal damage, including renal AL amyloidosis (5, 7). The main clinical manifestation of renal AL amyloidosis was nephrotic syndrome without marked hematuria and hypertension, and the pathological feature of renal tissue was the deposition of amorphous substances in glomeruli, interstitium and small arterial walls. These amorphous substances were composed of monoclonal light chain and were positive for Congo red staining. Producing an apple-green birefringence under a polarized light. Monoclonal λ light chain is the most common immunoglobulin involved. This patient’s clinicopathological features are completely consistent with renal AL amyloidosis.

### 3.2 Pathological spectrum of WM-related kidney disease

The pathological spectrum of WM-related kidney disease is diverse, encompassing glomerular, tubulointerstitial, and vascular lesions (7). In this case, the diagnosis of AL-type renal amyloidosis was confirmed by kidney biopsy, a pathological type consistently identified as one of the most common forms of WM-related kidney disease in multiple large cohort studies. A multicenter retrospective study by Chauvet et al. in France included 35 patients with IgM monoclonal gammopathy-related kidney disease, among whom AL-type renal amyloidosis was identified in 11 cases (31%), representing the most common type of kidney lesion in this cohort (8). A retrospective study conducted by the Mayo Clinic showed that among 57 patients with IgM-related B-cell lymphoproliferative disorders who underwent kidney biopsy, monoclonal gammopathy-related kidney lesions were identified in 47 cases (82%), with amyloid-related glomerulopathy observed in 19 patients (33% of the total cohort), likewise representing the most common pathological type (10). Vos et al. analyzed 1,391 patients with WM and identified 44 cases of biopsy-proven WM-related nephropathy, of which AL-type renal amyloidosis accounted for 25% (9). These data indicate that AL-type renal amyloidosis plays a significant role in WM-related kidney disease and warrants high clinical suspicion.

### 3.3 Treatment strategies for WM-related nephropathy

#### 3.3.1 Treatment goals

The treatment goal for WM-associated AL renal amyloidosis is to achieve rapid and deep elimination of the pathogenic light chains, thereby minimizing end-organ damage (15). The BSH guideline clearly states that organ involvement in AL (IgM-related) amyloidosis is a clear indication for treatment initiation, with the therapeutic goal being rapid and deep reduction of light chain levels (11).

#### 3.3.2 Chemoimmunotherapy

The patient’s WM and associated renal AL amyloidosis require treatment. The patient in this case tested positive for the *MYD88* mutation, while the *CXCR4* mutation status remained unknown due to a lack of testing. The key driver mutations in WM include *MYD88* and *CXCR4*, with the former being present in 95%-97% of patients and the latter in 30%-40% of patients. Both mutations jointly influence disease phenotype and response to targeted therapies (16–18).In recent years, genomics-based treatment strategies for WM have gained increasing attention. In 2020, Treon and colleagues (19) proposed a genomic-based treatment strategy for WM, that is, selecting therapeutic drugs according to the mutation status of *MYD88* and *CXCR4*. This strategy was further revised in 2025 (20). However, the mutation status of the *CXCR4* gene in the patient was unclear as no testing was conducted. How should the therapeutic regimen be selected in this case? After multidisciplinary consultation, it was decided to use the BR regimen for this patient, as this regimen is effective across different *MYD88* and *CXCR4* genotypes in WM (11, 12, 20).

Additionally, the BR regimen has also demonstrated encouraging efficacy in WM- related AL amyloidosis. Manwani et al. (21) reported that among 27 patients with IgM-related AL amyloidosis treated with BR, the overall hematologic response rate was 59%, with a very good partial response (VGPR) rate of 37%. Milani et al. (22) further confirmed in a study of 122 patients with AL amyloidosis that the IgM-AL subgroup receiving rituximab-containing BR achieved a hematologic response rate of 58%.These data indicate that BR is not only effective across a broad range of genotypes in WM but also shows clear efficacy in the specific subgroup of WM-associated AL amyloidosis. Therefore, although the *CXCR4* mutation status of this patient remained unclear, the BR regimen was selected as the first-line treatment after multidisciplinary discussion, based on its efficacy across various *MYD88* and *CXCR4* genotypes and its favorable outcomes in WM-associated AL amyloidosis. This decision reflects a prudent choice of a broadly effective regimen in the context of incomplete genetic information, and is consistent with current guideline recommendations for the treatment of WM-associated AL amyloidosis (11, 23). After three cycles of treatment with BR regimen, the patient achieved significant hematologic response, but proteinuria only decreased slightly. In fact, the treatment of renal AL amyloidosis is a huge challenge, and the substantial improvement of proteinuria typically requires prolonged therapeutic intervention. However, continued use of the BR regimen may result in severe adverse reactions, including infections. Unfortunately, during subsequent treatment, the patient did develop a serious lung infection, ultimately leading to death.

The advantage of the BR regimen lies in its time-limited therapy, which provides deep and durable responses and extends treatment-free intervals. However, they also share common limitations, including the potential to cause cytopenias and an increased risk of infections during and after treatment (24, 25). In a retrospective analysis of older patients with indolent non-Hodgkin lymphoma treated with bendamustine, Fung et al. (24) found that bendamustine exposure was associated with an increased risk of both common and opportunistic infections. Bendamustine can cause prolonged T-cell depletion, which may increase the risk of opportunistic infections and potentially compromise the efficacy of subsequent T-cell-dependent immunotherapies (25). As a key component of the BR regimen, the aforementioned adverse effects of bendamustine may contribute to an increased infectious risk associated with this regimen. Infection prevention strategies are critical during BR therapy. The NCCN guidelines (12) note that prophylaxis against Pneumocystis jirovecii pneumonia (PJP) should be considered for patients receiving bendamustine/rituximabc. Similarly, the BSH guidelines recommend (11) PJP prophylaxis for patients receiving intensive immunosuppressive therapy, and suggest immunoglobulin replacement therapy for patients with secondary hypogammaglobulinemia who experience recurrent infections despite antimicrobial prophylaxis.

Lessons should be learned from the treatment of this patient. During the treatment of BR regimen, it is essential to closely monitor the changes of blood cells and immune function of patients, strengthen comprehensive supportive therapy, and implement antimicrobial prophylaxis when necessary.

#### 3.3.3 Proteasome inhibitors

Proteasome inhibitors are highly active in WM, and the combination of bortezomib, dexamethasone, and rituximab (BDR) has been extensively studied. In a large retrospective real-world analysis (26) conducted at eight tertiary medical centers in France, a total of 87 patients with symptomatic WM were included. The BDR regimen achieved an overall response rate of 88% and a major response rate of 75%. The median time to best response was 9 months, and the median event-free survival for the entire cohort was 33 months. BDR demonstrated favorable activity in patients with both double-mutant and wild-type *MYD88/CXCR4* status, positioning it as an alternative to BTK inhibitors. Regarding safety, peripheral neuropathy was the most prominent toxicity, with an incidence of 23% for any grade and 7% for grade 3–4 events, leading to treatment discontinuation. Infectious events occurred in five patients, including pneumonia and pneumococcal meningitis. Notably, among the 21 patients who died from non-WM-related causes, 8 (38%) died from infections, including COVID-19, cryptococcal meningitis, pneumococcal sepsis, and Pneumocystis jirovecii pneumonia, highlighting the importance of implementing infection prevention strategies during and after proteasome inhibitor-based therapy. In a phase II trial of previously untreated patients with WM (27), the BDR regimen demonstrated rapid responses and a high overall response rate. However, reversible peripheral neuropathy was the most common toxicity associated with this regimen, leading to premature discontinuation of bortezomib in 61% of patients. In addition, herpes zoster reactivation was frequently observed in patients without antiviral prophylaxis, and prophylaxis for herpes zoster is recommended when using BDR.

The combination of BDR has also shown favorable efficacy in WM-associated AL amyloidosis. Proteasome inhibitor-based regimens can rapidly reduce immunoglobulin M (IgM) levels, minimize the risk of IgM flare, and avoid significant short- or long-term myelotoxicity, thereby providing a chemotherapy-free treatment option. For patients with AL amyloidosis, cryoglobulinemia, cold agglutinin disease, or those with high IgM levels who are unable to receive BTK inhibitors or bendamustine plus rituximab due to cardiovascular comorbidities or myelotoxicity, bortezomib- based regimens such as BDR are preferred treatment options (28, 29). Neuropathy is the predominant toxicity observed with bortezomib-based regimens (12) Therefore, when selecting a bortezomib-based regimen, treatment should be tailored to individual patient characteristics and the toxicity profile should be carefully balanced.

#### 3.3.4 Brutontyrosine kinase inhibitors

BTK inhibitors (BTKis) have become a cornerstone in the treatment of Waldenström macroglobulinemia (WM), demonstrating high efficacy in both treatment-naïve and relapsed/refractory patients. Covalent BTK inhibitors, including ibrutinib, acalabrutinib, and zanubrutinib, induce apoptosis in WM cells by disrupting downstream NF-κB signaling, particularly in tumors harboring the *MYD88*^L265P^ mutation (30). Zanubrutinib, a more selective covalent BTK inhibitor, has shown superior depth of response and favorable efficacy across different *MYD88* and *CXCR4* genotypes, with a very good partial response (VGPR) rate of 45% and a 3-year progression-free survival (PFS) rate of 81% in a phase II study (31). Although BTK inhibitors such as ibrutinib and zanubrutinib have established efficacy in WM, their use in WM-associated AL amyloidosis requires caution. Pika et al. (32) reported suboptimal efficacy, poor tolerability, short survival, and high toxicity rates in patients with IgM-related amyloidosis treated with ibrutinib. The 11th International Workshop on Waldenström’s Macroglobulinemia (IWWM-11) consensus (23) clearly states that BTKis are not recommended as first-line therapy for WM-associated AL amyloidosis and are contraindicated in patients with concomitant cardiac amyloidosis. The toxicity profile of BTKis also warrants attention. Hematologic adverse events, particularly neutropenia, occur across all BTK inhibitors (10–20%). Ibrutinib is associated with higher risks of atrial fibrillation (up to 13-20%), hypertension (grade ≥3 incidence 10-13%), and major bleeding due to its off-target inhibition of kinases such as ITK and TEC. In contrast, newer-generation agents such as zanubrutinib and acalabrutinib offer improved cardiovascular safety, with atrial fibrillation rates of 2-5% and grade ≥3 hypertension in 3-6% (30).

#### 3.3.5 Transplantation in WM

For eligible patients, particularly those with WM-associated AL amyloidosis, autologous stem cell transplantation (ASCT) represents an important therapeutic option. Sidiqi et al. (33) reported outcomes in 38 patients with WM-associated AL amyloidosis who underwent ASCT, demonstrating an overall hematologic response rate of 92%, with 76% achieving at least a very good partial response (≥VGPR), a renal response rate of 65%, and a 100-day treatment-related mortality rate of 5%. The 11th International Workshop on Waldenström’s Macroglobulinemia (IWWM-11) consensus supports ASCT as a first-line or consolidation strategy for eligible patients (15). These findings underscore the importance of early consideration of transplantation in suitable candidates.

However, patient selection is crucial. For younger, fit patients who may be eligible for ASCT in the future, the use of stem cell-toxic agents (such as bendamustine or purine analogs) should be avoided in first-line therapy to reduce the risk of stem cell collection failure (34).The role of allogeneic stem cell transplantation (allo-SCT) in WM has become more controversial, particularly in the era of novel agents. Due to a high non-relapse mortality (NRM) of approximately 30%, its use is limited to highly selected patients, typically those who have progressed after immunochemotherapy and BTK inhibitor therapy (35, 36).

#### 3.3.6 Considerations for treatment selection

Since WM is currently considered incurable, optimizing patients’ quality of life has become an important therapeutic paradigm. The most effective way to achieve this goal is to strike a balance between effective disease control and minimizing treatment-related toxicity and adverse effects (30).The optimal initial treatment regimen should be individualized based on patient-specific factors, including patient characteristics (such as performance status, baseline comorbidities, and preference for fixed treatment duration), disease-related factors (such as disease burden, presence of cytopenias, and immunoglobulin M levels), and mutation status (such as the presence of *CXCR4* and *MYD88* mutations).

## 4 Conclusion

This paper reports an extremely rare case of WM-associated renal AL amyloidosis, which provides us with the following valuable experiences and lessons: first, the serum and urine M-FLC should be tested in WM patients, as it exists in WM at a high proportion and can independently cause MGP-RD, including renal AL amyloidosis. Second, when the genotypes of *MYD88* and *CXCR4* of WM patients are not yet clear, especially when accompanied by AL amyloidosis, it is reasonable to choose BR regimen as the first-line treatment. However, renal AL amyloidosis usually requires relatively long-term treatment. During the treatment process, it is essential to closely monitor the adverse effects of the BR regimen, particularly infections, which can lead to serious poor consequences. The emergence of various novel treatment options has greatly improved the survival rate of patients with WM, making it increasingly important to balance the goal of prolonging survival with minimizing treatment-related toxicity. Several issues remain to be addressed in the future: first, there is a lack of prospective clinical trials specifically targeting this patient population; second, the optimal timing and duration of treatment for WM-related nephropathy have not yet been standardized. Future multicenter, prospective studies are needed to further optimize treatment strategies and improve patient outcomes.

## 5 Patient perspective

The patient was a 71-year-old woman whose life was significantly impacted by a 10-month history of progressive bilateral lower limb edema and profound fatigue, which greatly limited her daily activities and quality of life. Following a comprehensive diagnostic workup that established the diagnosis of Waldenström macroglobulinemia-associated renal AL amyloidosis, she and her family were thoroughly counseled regarding the proposed bendamustine plus rituximab (BR) regimen. The potential benefits of this treatment in controlling the underlying lymphoplasmacytic lymphoma and the associated risks, particularly immunosuppression and infection, were clearly explained and understood. The treatment goal of reducing the monoclonal IgM burden and preserving renal function was mutually acknowledged. She tolerated the first three cycles of BR therapy well. During this period, a marked decrease in serum IgM levels was observed, indicating a favorable hematologic response. Concurrently, the treatment yielded partial renal benefit. The patient and her family expressed appreciation and found comfort in the positive hematologic and renal responses achieved with the therapy. However, due to her poor overall clinical condition and compromised health status, she unfortunately developed a severe pulmonary infection during the subsequent phase of treatment. Despite receiving intensive medical care in the hospital, her condition deteriorated rapidly, and she ultimately succumbed to the infection. This tragic outcome underscores the critical importance of vigilant monitoring for and aggressive prophylaxis against infectious complications when administering intensive chemoimmunotherapy to clinically vulnerable patients.

## Data Availability

The original contributions presented in the study are included in the article/supplementary material. Further inquiries can be directed to the corresponding authors.
